# Preliminary Case Series of the Worth Warrior Mobile App for Young People With Low Self-Esteem and Mild Eating Disorders: Pre– and Post–Follow-Up Study

**DOI:** 10.2196/79770

**Published:** 2026-01-20

**Authors:** Rachel Edwards, Nihara Krause

**Affiliations:** 1 stem4, stem4, Connect House, 133-137 Alexandra Road, Wimbledon, London, SW19 7JY, United Kingdom, 44 7956396375

**Keywords:** mental health, eating disorders, body image, self-esteem, mobile phone app, cognitive behavioral therapy, CBT-E, digital tool, young people, mobile phone

## Abstract

**Background:**

Eating difficulties are increasingly prevalent among young people, yet service capacity remains limited. Digital interventions may provide accessible, scalable support, particularly for those with mild, subthreshold, or early-stage symptoms who do not meet criteria for specialist care. Low self-esteem is widely recognized as a key psychological risk factor in the onset and persistence of eating disorders, and negative self-evaluation, particularly around body image and social acceptance, can heighten vulnerability to the maladaptive thoughts and behaviors seen in conditions such as anorexia nervosa, bulimia nervosa, and binge-eating disorder. Clarifying this relationship is essential for developing effective prevention and early intervention strategies.

**Objective:**

This pilot case series reports on 5 individuals aged 19-25 (mean 22, SD 2.19) years with mild eating disorders who used the Worth Warrior app, a mobile intervention incorporating principles of enhanced cognitive behavioral therapy strategies targeting low self-esteem, body image concerns, and disordered eating behaviors.

**Methods:**

An uncontrolled 3-phase design (baseline, post–app familiarization, and follow-up) was used. Participants completed standardized self-report tools, including the Eating Disorder Examination Questionnaire and Rosenberg Self-Esteem Scale. Feedback on usability, acceptability, and safety was also collected via online questionnaires.

**Results:**

Outcome measures at follow-up showed improvements in eating disorder symptomatology in 3/5 cases, and in self-esteem in 4/5 cases; those with milder symptomatology indicated the most benefit. Reductions in eating concerns, weight concerns, and related behaviors were observed in most, though not all, cases. Participants valued interactive enhanced cognitive behavioral therapy features and journaling functions, while noting areas for improvement such as reminders and incentives for use and preventions for maladaptive use of the free-text facilities.

**Conclusions:**

Findings suggest the Worth Warrior app may be suited as an acceptable and effective standalone tool for individuals with mild eating disorder symptoms, and used as an adjunct to traditional treatment alongside clinician supervision for those with more severe presentations to promote the greatest patient safety. These exploratory case study findings suggest that the app has the potential to support improvements in self-esteem and mild eating disorder symptomatology; however, as a preliminary case series, these results are not generalizable but provide a foundation for larger, controlled studies of digital early intervention.

## Introduction

### Background

Eating disorders are common and distressing mental health conditions with significant psychological, physical, social, and economic impacts. An individual with an eating disorder controls their food intake in some way to manage difficulties such as strong emotions, and these disorders involve negative thoughts and behaviors about oneself, eating, and physical appearance, often resulting in food restriction and/or binging, as well as associated behaviors such as excessive exercising [1-3]. The most common forms of eating disorder are anorexia nervosa, bulimia, and binge eating disorder [1].

### The Prevalence of Possible Eating Problems and Clinical Eating Disorders in Young People

An estimated 1.25 million people in the United Kingdom (UK) are currently living with an eating disorder, a considerable proportion of whom are younger than the age of 25 years [4,5]. The number of referrals for eating disorders to National Health Service (NHS) child and adolescent mental health services in England has almost doubled from prepandemic levels, and these rates have remained elevated since, with those younger than 25 years making up 48% of total hospital admissions for eating disorders [5-8]. In 2023, a total of 3% of those aged 11‐16 years, 13% of those aged 17‐19 years, and 6% of those aged 20‐25 years were identified as having an eating disorder [7]. This all points to a persistent and rising tide of eating disorder problems among young people in the United Kingdom.

Furthermore, the prevalence of “possible eating problems” among young people is even higher [7,9]. A possible eating problem typically refers to behaviors, attitudes, or symptoms associated with disordered eating that may not meet the full clinical criteria for an eating disorder, but are nonetheless concerning and can impair health and well-being. In 2023, almost 60% of those aged 17‐25 years and 12% of those aged 11‐16 years had a possible eating problem [8]. This is of concern because subthreshold cases represent a vulnerable group at heightened risk of progressing to full-syndrome eating disorders.

### Self-Esteem, Body Dissatisfaction, and the Development of Eating Disorders in Young People

The etiology of an eating disorder is complex, with overlapping biopsychosocial and cultural factors implicated in its onset and development [10]. Research indicates that the mean age of onset for eating disorders such as anorexia nervosa and bulimia nervosa is between about 15 and 19 years, while binge-eating disorder tends to emerge slightly later, at around 23 and 24 years [1,3,11]. These disorders are often triggered and/or maintained by biopsychosocial factors that can include sociocultural pressures, puberty and changing bodies, low self-esteem, and body dissatisfaction [12-14].

Indeed, low self-esteem and body dissatisfaction are consistently identified as significant, transdiagnostic vulnerability factors for and predictors of the onset and maintenance of eating disorders [15-20]. “Self-esteem” is defined here as a person’s subjective evaluation of themselves and their worth [21,22], and individuals with chronically low self-esteem may attempt to regulate feelings of inadequacy and negative self-evaluation through control of overeating, weight, and shape [23]—all factors involved in eating disorders. Furthermore, longitudinal studies show that low self-esteem in adolescence predicts later onset of disordered eating behaviors [24,25].

Moreover, low self-esteem is closely linked with body dissatisfaction, which is a form of body image involving a negative attitude toward the appearance of one’s body [26] and one of the strongest predictors of eating pathology [27,28]. “Body image” refers to a dynamic and multidimensional construct, incorporating a person’s cognitive, affective, and evaluative perceptions of their own body and how it appears [29-31]. When a person places excessive value on their weight and shape and is dissatisfied with their body, they too may develop disordered eating behaviors and pathologies to try and regulate or “improve” this body image [19,29].

Importantly, these factors have also been shown to be reciprocally related, such that low self-esteem can enhance body dissatisfaction and vice versa [15,32,33]. For example, of particular interest in eating pathology is appearance-contingent self-esteem, wherein a person’s self-esteem is dependent upon their evaluation of their physical appearance [34,35]. When individuals base their self-esteem predominantly on appearance, they are more likely to experience body dissatisfaction, which in turn contributes to lower self-esteem and poor mental health outcomes, including anxiety, depression, and disordered eating (eg, Adams et al [36], 2017; McLean and Paxton [17], 2019; Mora et al [18], 2017). This process creates a cycle where body dissatisfaction undermines broader self-value, increasing the risk of restrictive or compensatory behaviors. Clinical models, such as Fairburn’s transdiagnostic theory, therefore identify low self-esteem as a maintaining mechanism that interacts with perfectionism and self-criticism, thereby perpetuating the cycle of dietary restraint, binge eating, and compensatory behaviors [23].

Consequently, addressing body dissatisfaction and self-esteem may be an important preventive and early intervention target, particularly with regard to building self-esteem that is not dependent on weight or body image. Evidence indeed suggests that therapeutic approaches that target self-esteem and body dissatisfaction, such as enhanced cognitive behavioral therapy (CBT-E), are effective in reducing eating disorder symptoms [37,38]. Interventions that foster body acceptance, promote diverse standards of beauty, and encourage self-esteem rooted in internal qualities rather than appearance have been shown to mitigate the aforementioned risks of appearance-contingent self-esteem and support healthier psychological development and can therefore be a valuable inclusion in the prevention and treatment of eating disorders [39-41]. As part of this, therefore, identifying and supporting subthreshold cases is crucial.

### The Importance and Difficulty of Early Intervention and Long-Term Treatment

With varied and serious health risks associated with eating disorders, including mortality, growth delay, hypovolemia, and relapse, adequate and timely treatment is essential, and prevention and early intervention are ideal [12,42]. Intervening during the early stages of an eating disorder has multiple benefits, including preventing functional deterioration, neuroadaptation, and habitual behavior patterns, as well as the challenges associated with prolonged illness [43]. Early intervention can reduce progression to severe disorders, decrease health care use, and improve quality of life, while also generating economic savings by offsetting the high health, social, and economic costs associated with the illness [44-47].

Despite this, delays in treatment are common, with many young people in the United Kingdom being left on long waiting lists or unable to access care, and NHS targets for timely treatment are not currently being met [5,48]. With stretched services having to prioritize higher-severity cases, the duration of untreated eating disorders has been estimated at around 3 and a half years [49]. These treatment delays are largely due to demand rapidly outpacing treatment capacity, service funding being cut, and early-stage disorders often going undetected or untreated for long periods due to stigma, lack of access to care, and insufficient service provision [4,46,50]. Unfortunately, treatment delays are associated with poorer outcomes, with the best prognosis found in young people with a short duration of illness [51-54], and this is particularly concerning given that anorexia nervosa has the highest mortality rate of any psychiatric disorder, with mortality risk in those aged 15‐24 years exceeding that of other major adolescent illnesses such as asthma or type-1 diabetes [48,55,56].

Lastly, these issues are further compounded by the fact that eating disorders are complex, chronic conditions that frequently require extensive and long-term treatment. Recovery often takes several years, and relapse rates are high, and guidelines emphasize the need for sustained, multidisciplinary approaches [1,48,57]. While early intervention clearly improves outcomes, the reality of long-term care further compounds capacity pressures, underlining an urgent need for scalable and accessible solutions to this growing problem.

### Mobile Health Apps for Eating Disorders and Self-Help Treatment

This increased demand, coupled with delays in accessing care, therefore highlights the need for expanded capacity for effective, accessible support and early intervention for eating disorders in young people. Digital interventions can thus play a potential role in delivering timely care at scale by enabling young people to manage symptoms before they become severe, serving as an adjunct while awaiting or receiving treatment, or helping patients to stay well and avoid relapse following treatment [58-60]. In particular, given that approximately 96% of adolescents aged 12‐17 years own or have access to a mobile phone [61], mobile health apps could increase access to psychological therapies by providing treatment-informed techniques to a wide audience in a digital format. These applications also offer additional advantages to traditional in-person support, such as face-to-face cognitive behavioral therapy, including scalability at low marginal cost, capacity to reach underserved populations (eg, rural or low-income groups), and the potential to reduce stigma and facilitate early triage for specialist care [59,62,63].

There are some existing mobile health apps for the management of eating disorders, which typically aim to provide either information, self-monitoring, assessment, or support to users, or a combination [60]. However, evidence for the safety, efficacy, and effectiveness of the majority of such tools remains limited at best [64]. Many existing apps lack grounding in evidence-based frameworks, incorporate minimal user involvement in their design, and omit robust safety protocols [64-66]. The scope of these apps also varies widely; in a systematic clinical appraisal by Fairburn and Rothwell (2015) [64] assessing 39 such apps, only one-third provided psychoeducational information to users, 60% purported to provide support or advice, and just 10% facilitated self-monitoring of eating habits. Given the complexity of eating disorders and the need for a multidisciplinary approach to treatment, a comprehensive intervention would ideally incorporate all of these elements. More concerningly, 85% of those who provided psychoeducational information to users and 70% of those who provided support or advice were found to provide either unreliable, misleading, or harmful content. Furthermore, the unique potential of smartphone technologies—such as ecological momentary interventions and automated self-monitoring—remains largely underexploited in existing tools, restricting the advantages these digital interventions could offer over traditional treatment approaches [65].

Results from a systematic review by Wasil et al [67], 2021, do at least suggest that most active app use focuses on a select few apps for eating disorder management that are evidence-based. However, none of these most prominent apps appear to focus specifically on addressing the aforementioned relationship between body image, self-esteem, and eating disorders that can be critical to both the development and maintenance of these illnesses, nor are they designed and intended for children and young people specifically. Many also have some or all features behind a paywall and/or require clinician involvement, all of which can restrict the accessibility and scalability advantages that can be afforded by apps.

### The Worth Warrior Mobile App Intervention

In response to this, the Worth Warrior mobile app is a clinician-developed intervention intended to address this gap in the existing digital mental health landscape by providing young people at risk of developing eating disorders with access to evidence-based support that addresses the transdiagnostic risk and maintenance factors of low self-esteem and body dissatisfaction. Its applicability spans mild forms of eating disorders including anorexia nervosa, bulimia nervosa, binge-eating disorder, and avoidant/restrictive food intake disorder, reflecting the transdiagnostic nature of eating disorders.

It is therefore designed to support young people who have low self-esteem, body dissatisfaction, and mild eating difficulties, drawing on principles of CBT-E. In particular, the app focuses on breaking the aforementioned cycle of poor body image and low self-esteem that leads to eating-related thoughts and behaviors, delivers psychoeducation, and helps to monitor and change negative or critical thoughts, eating-related behaviors, the daily impact of negative self-image, and emotional regulation—all factors that can contribute to a young person’s eating behavior and negative self-esteem.

The app seeks to address limitations in the existing digital health landscape in its evidence-based focus and comprehensive CBT-E components, its leveraging of smartphone technology (such as automatically populated self-monitoring and both personalizable and interactive activities), and its prompts to seek further help when times of high risk are identified [65,66,68]. Its development also involved collaboration with young people and clinical experts, with iterative safety reviews, mitigating the aforementioned risk of providing potentially harmful information.

However, evidence is required to demonstrate the app’s effectiveness and safety.

### This Study

This paper presents a preliminary evaluation of the Worth Warrior app in the form of a case series of 5 young people with mild eating disorders who used the app for 7 weeks, with a focus on any clinical change in their eating disorder–related thoughts and behaviors and self-esteem throughout the intervention period, as well as their reception of the app itself (specifically its usability, acceptability, and safety). “Mild” eating disorders were identified as self-reported eating difficulties that were subthreshold, or above but without a formal diagnosis, professional treatment, or indicators of greater severity such as previous hospital admission.

Due to the small number of participants completing all phases of this study (expanded upon later), this study has been written up qualitatively as a case series. Case reports and series can be a crucial first step in developing evidence of the impact of a new treatment intervention, prompting further research and highlighting questions to address, as well as centering on the patients’ perspectives and experiences [69].

## Method

### Study Aims

This preliminary evaluation of the Worth Warrior app aims to present a case series of 5 individuals’ experiences of using the app over 7 weeks, with a focus on any clinical changes in eating disorder and/or self-esteem levels over the intervention period, along with their perceived usability, acceptability, and safety of the app. The objective of this research was to ascertain whether these individuals found the app useful in managing and/or reducing their mild eating disorders and low self-esteem, to contribute toward preliminary evidence of the effectiveness of the Worth Warrior app as a tool for young people with such difficulties.

### Intervention Design and Components

#### About the Intervention

The Worth Warrior smartphone app is a clinician-developed, standalone tool for individuals aged 12 years and older who are experiencing self-esteem issues or body image concerns, and who may be at risk of developing, or be at the early stage of, disordered eating behaviors. The app provides structured interactive modules that address cognitive, behavioral, and emotional processes associated with eating-related difficulties, based on the principles of CBT-E.

It is intended for multiple groups, including (1) young people who do not meet threshold criteria for specialist treatment, (2) those awaiting specialist services, (3) individuals receiving treatment where the app may serve as an adjunct, and (4) those posttreatment seeking to maintain progress and prevent relapse. It can also provide key support to those young people aged 16‐25 years who meet First Episode Rapid Early Intervention for Eating Disorders criteria. It is important to note, however, that the intervention is not intended as a substitute for assessment and intervention by a mental health professional.

#### CBT-E Components

The CBT-E components of the app include (1) cognitive restructuring of negative self-thoughts (“change the story”), (2) behavioral modification and response prevention (“change the action”), (3) development of alternative emotional regulation strategies (“change the emotion”), and (4) body image–focused exercises aimed at challenging distorted assumptions (“change the way I view my body”).

**Figure 1.**
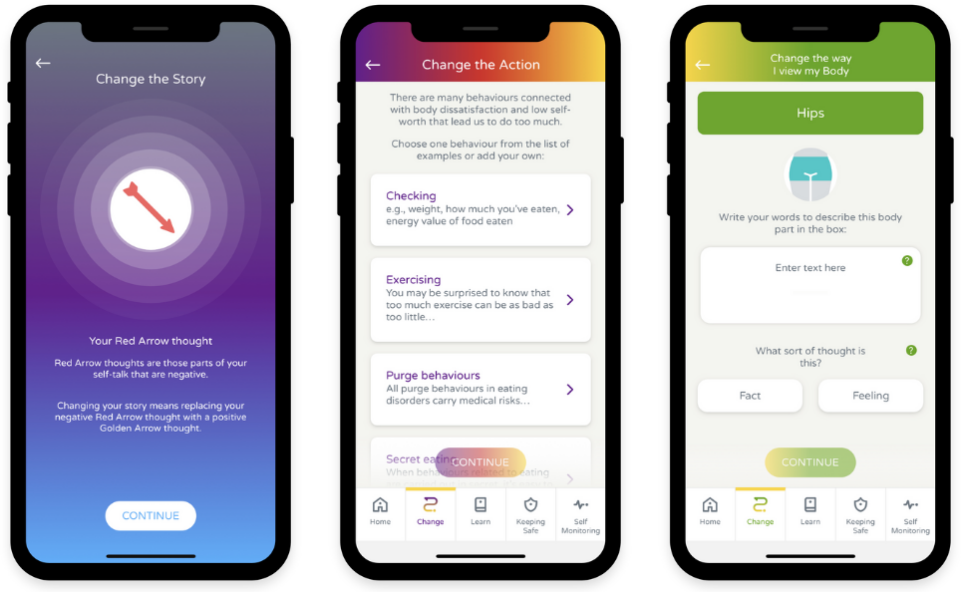
Example screens in the “change the…” sections of the Worth Warrior app, the digital intervention used in this preliminary case series for young people with low self-esteem and mild eating disorders.

See Figure 1 for example screens of some of these sections of the app.

The app does not, however, incorporate the tracking component of traditional CBT-E in which the person monitors their nutrition and weight, as it was determined that this would not be suitable in a tool that can be used independently, and was also a preference expressed by young people during co-design. Instead, the app offers psychoeducation on food and eating, along with perfectionism, body dissatisfaction, and conditions such as body dysmorphic disorder, avoidant/restrictive food intake disorder, and eating difficulties associated with autism spectrum conditions.

**Figure 2.**
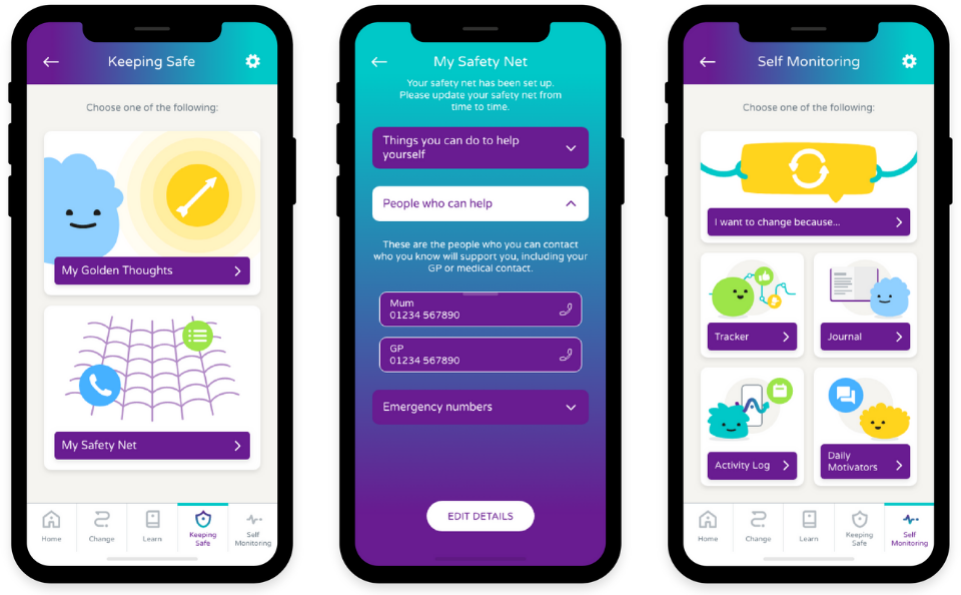
Example screens of the keeping safe, safety net, and self-monitoring sections of the Worth Warrior app, the digital intervention used in this preliminary case series for young people with low self-esteem and mild eating disorders. GP: general practitioner.

Risk management is supported through a “keeping safe” section (see Figure 2 for example screens), which allows users to identify supportive strategies, contacts, and personalized positive statements. Self-monitoring features enable users to track progress, journal thoughts and feelings, and access motivational prompts.

#### Co-Design

Worth Warrior was designed to include age-appropriate design elements for adolescents and young adults, and co-design occurred at multiple stages in development. Initial ideas on the content of the app, design, and tone of voice were obtained by involving 5 young people with lived experience and 1 parent as well as a group of 10 young people aged 15‐17 years from the community. All individuals involved were diverse in terms of gender and ethnicity.

The groups were approached again once a minimal viable product was designed for further feedback on user journeys and to provide feedback on challenges and strengths. A total of 8 young people in the community then tested the app and provided feedback. Additionally, 4 young people had early stage body image issues. Moreover, 12 young people with lived experience of a variety of mental health difficulties were involved in the research design. Clinicians comprising 4 clinical psychologists specializing in eating disorders, 2 child and adolescent psychiatrists, a psychotherapist, and an eating disorder researcher all contributed to feedback on the app and attended 2 clinical hazard workshops for input and feedback.

#### Safety and Regulatory Considerations

The app is a noninvasive, noncontact digital health intervention with flexible usage patterns. While no physical risks are associated with its use, clinical oversight is recommended in moderate presentations. It is not intended as a substitute for professional assessment or treatment; users are directed to medical consultation where necessary. The app complies with clinical safety standards (eg, NHS DCB0129) and prioritizes data protection through minimal data collection and privacy safeguards. This is particularly pertinent considering that the 2 most common perceived barriers to eHealth interventions for eating disorders found by Linardon et al [63], 2021, are concerns regarding data privacy and information accuracy.

### Evaluation Design and Participants

#### Overview

This study applied a 3-stage, pre– and post–follow-up, case series design.

#### Sample, Recruitment, and Consent

Participants were recruited using a combination of sampling methods with the intention of involving a diverse and representative sample. This included reaching out to several national eating disorder organizations (Beat, National Centre for Eating Disorders, SWEDA, and First Steps ED), specialist eating disorder services (Birmingham Women’s and Children’s NHS Foundation Trust), leaflets and posters in national community centers, and universities, who shared about this study’s recruitment with their community groups and/or on their website. The recruitment advertisement sent to these organizations can be found in the Multimedia Appendices (Multimedia Appendix 1). Additionally, this was shared via stem4’s (the app developer’s) website and existing social media networks.

Messaging in these advertisements included, “Do you have low self-esteem and think you have a sub-clinical or early stage eating disorder?” A 13-item screening tool, developed for the purposes of this study, was used to assess the young person’s eligibility (Multimedia Appendix 2). Participants also needed to sign a statement confirming their fitness to participate.

Participant inclusion criteria included (1) young people aged between 17‐25 years, (2) those who presented with body image issues or eating-related difficulties (assessed via the screening tool; Multimedia Appendix 2), and (3) were not currently receiving treatment from secondary mental health services.

“Mild” eating disorders in this study were identified as those scoring below the Eating Disorder Examination Questionnaire (EDE-Q) clinical threshold, or above but without a formal diagnosis, professional treatment, or indicators of greater severity such as previous hospital admission. This was determined via responses to the screening tool and EDE-Q scores obtained from the baseline questionnaire.

See Multimedia Appendix 3 for the full inclusion criteria and each corresponding screening question.

Exclusion criteria included anyone at high risk (eg, with active suicidal ideation or considering or planning suicide) or currently experiencing a crisis or episode of illness. This also excluded those diagnosed with a severe and enduring mental health problem, such as psychosis, or a significant learning disability, or individuals who may be misusing alcohol or substances which may have hampered their use of the app.

Eligible participants were given an information sheet informing them of this study’s details, risks, and benefits before being asked to complete an informed consent form. Only those who provided their informed consent went on to enter this study.

#### Evaluation Procedure

##### Overview

Textbox 1 shows this study’s procedure. We also consulted with 8 people (aged between 14 and 17 years) to provide feedback on this study’s proposal and procedure regarding their preferences concerning web-based data collection.

Textbox 1. **Procedure for this preliminary case series pilot study of the Worth Warrior app for young people with low self-esteem and mild eating disorders.**

**Recruitment and consent**

- Advertise the Worth Warrior app evaluation and invite young people to participate
- Check the eligibility of young people interested by using the screen questionnaire
- Obtain informed consent from young people

**Baseline and app familiarization (stage 1)**

- First questionnaire with the consenting young person, if preferred
- Completion of all quantitative measures via an online link
- Downloading the app with instructions for use
- Familiarization with the app over 1 week

**Postfamiliarization review (1 week following baseline; stage 2)**

- Completion of the measures and questionnaires via online link
- Shorter questionnaire to review the app to assess safety and any problems with use, if preferred
- Decision of young people to proceed with using the app and taking part in the evaluation

**Use of the Worth Warrior app for 6 weeks**

- Participant to use the app when needed over the next 6 weeks

**Follow-up (stage 3)**

- Participants complete outcome measures online for the last time
- Final questionnaire to assess the app’s safety, usability, and acceptability, and whether they contacted someone for help

The evaluation procedure comprised three data collection stages: (1) baseline and app introduction (stage 1), (2) post–app familiarization of one week (stage 2), and (3) follow-up after six weeks (stage 3).

##### Baseline (Stage 1)

Participants were provided with online instructions on using the Worth Warrior app and the evaluation procedure. Once their eligibility was determined and informed consent was given, they were asked to complete 3 baseline clinical assessments online to measure symptoms of self-esteem and eating disorder symptomatology (details of these questionnaires are provided below). Participants also completed a brief, standard questionnaire to collect data on their demographic characteristics.

Participants were asked to download the Worth Warrior app and familiarize themselves with it over the next week. Alongside obtaining baseline measures, this familiarization period was to manage risk and ensure participants’ safety until they became fully versed in using the app, were aware of signs and symptoms to look out for, and had input information in their “safety net” section.

##### Post–App Familiarization (Stage 2)

One week after baseline, an online questionnaire was sent to participants to gauge their assessment of the app and any problems they may have had. The person could then decide whether they wished to continue with this study. If they chose to continue, they completed the standardized measures online. Participants could then use the app as often as they wanted over the following 6 weeks.

##### Follow-Up (Stage 3)

After 6 weeks, participants were asked to complete the clinical measures and user questionnaire for the final time.

##### Following Up on Nonresponders

At the postfamiliarization and follow-up stages, participants were contacted by email and asked to complete the follow-up questionnaires. If no response was received, the researchers made 2‐3 attempts to contact the participant. If no contact was made, the researchers assumed the participant no longer wished to take part in the evaluation.

### Data Collection

#### Clinical Outcome Measures

Two standardized clinical measures were used to assess the main study outcomes: mild symptoms of eating disorders and levels of self-esteem. These were the EDE-Q (version 6.0; [70]) and the Rosenberg Self-Esteem Scale [71].

#### About the EDE-Q

The EDE-Q is a 28-item self-reported questionnaire that assesses the range and severity of features associated with an eating disorder diagnosis, using 4 subscales (restraint, eating concern, shape concern, and weight concern), measures of behavior frequency, and a global score. It uses a 7-point Likert scale to assess the number of days and frequency of symptoms (ranging from “no days=0” to “everyday=6,” and “not at all=0,” to “markedly=6”). The scale can be used both to identify whether an individual can be considered to have a clinical eating disorder and the severity of any eating disorder–related psychopathology [72]. Its reliability and validity have been supported for both adults and adolescents, and in both clinical and nonclinical samples [72-75].

Global EDE-Q scores of 4 or more are generally accepted to indicate the presence of a clinical eating disorder [72].

#### Rosenberg Self-Esteem Scale

The Rosenberg Self-Esteem Scale is a 10-item self-report scale that measures global self-esteem. Each item is a statement relating to overall feelings of self-worth or self-acceptance, and items are rated using a 4-point scale ranging from “strongly agree” to “strongly disagree.” Individuals’ global scores from this scale can range from zero (the lowest possible score) to 30 (the highest possible), wherein scores within 15‐25 are generally considered to indicate “normal” levels of self-esteem, and those below 15 to indicate low self-esteem [76]. As such, the higher the score, the higher the level of self-esteem.

Again, the reliability and validity of this scale have been supported for both adults and adolescents [77-80].

#### Worth Warrior App User Questionnaire

A further questionnaire, developed for this study from a template from a previously published app evaluation [81], and administered online, involved 21 items to measure participants’ satisfaction with the Worth Warrior app (Multimedia Appendix 4). It used a combination of Likert rating scales, multiple choice questions, and open-ended questions with free-text fields to address the following domains: (1) usability—ease and frequency of use (seven items assessed this domain, such as “how easy was the app to use?” [1‐5 Likert scale]); (2) acceptability—participants’ opinions regarding the app and its usefulness, and how and why they chose to use it (ten items assessed this domain, such as “what features did you not like and why?” [free-text field]); and (3) safety—extent to which the app promoted positive outcomes (provided reassurance, reduced negative self-evaluation, and helped to contact someone when needed) and did not increase negative outcomes (four items assessed this domain, such as “did the app increase negative self-evaluation when you used it?” [free-text field]).

Participants were asked to complete this questionnaire at the postfamiliarization and follow-up time points.

All measures and questionnaires were entered onto an online platform, Survey Monkey, a link to which was sent to participants at baseline, post–app familiarization, and follow-up.

### Ethical Considerations

#### Ethical Approval and Informed Consent

Ethics approval for this study was obtained from the London—City and East Research Ethics Committee (United Kingdom; 22/HRA/1778), and all practices and procedures throughout the research process were in line with the stipulations of the Declaration of Helsinki. This included that participants needed to provide informed consent before commencing this study.

Participants were given a £30 (US $41.25) Amazon voucher as an honorarium upon completion of the final stage.

#### Participant Safeguarding

Participant exclusion criteria included anyone at high risk (eg, with active suicidal ideation or considering or planning suicide) or currently experiencing a crisis or episode of illness. Anyone who, at the initial screening, presented with increased risk was supported by the researchers to contact emergency numbers provided, including signposts to Beat (an eating disorder charity), NHS 111, and/or health services.

Additionally, as part of their informed consent, participants were told that the researchers could not offer counseling or any other form of support except for the Worth Warrior app for early stage eating disorders, which does not replace traditional treatment. They were also signposted to sources of help from the outset, and any take-up of these resources was noted where possible.

The inclusion of the app familiarization phase, as mentioned in the evaluation procedure, was also to facilitate participant safeguarding.

#### Data Protection and Privacy

To maintain confidentiality and anonymity, eligible and consenting participants were allocated an anonymous study ID number by a project administrator separate from the researchers. All participants’ data were anonymized, collected, and stored securely, and managed solely by the researchers.

#### Ongoing Monitoring and Risk Assessment

Throughout this study, participants self-reported their eating disorder symptoms and self-esteem levels at regular intervals, as well as indicators of relevant symptoms signaling worsening health. If they reported high scores or emerging new symptoms, they were encouraged to consult a general practitioner or, depending on the severity, seek more urgent help. After this study, participants were given resources on maintaining well-being.

stem4, the owners of the app, had a clinical safety protocol and hazard mitigation list that was managed and reviewed regularly, with clear timelines of response. Their clinical safety officer was available to respond to inquiries daily.

### Data Analysis

#### Overview

The demographic profile of participants and other quantitative data were described using frequencies, percentages, and means. Scores derived from the outcome measures were compared between baseline, postfamiliarization, and follow-up using Google Sheets and Looker Studio (Google LLC).

#### App User Questionnaire

Each subscale of the app user questionnaire was scored by averaging total ratings of the corresponding scales, resulting in a maximum score per subscale of 5.

Thematic analysis was conducted of the qualitative data from open-ended text responses, following the principles of Braun and Clarke [82], 2006, and, in particular, structured tabular thematic analysis as advocated by Robinson [83], 2021. Since this was a preliminary and exploratory evaluation of a new technology, an inductive approach was used for coding, in which codes and themes were generated naturally, from the bottom up, rather than guided by pre-existing theory. This decision was to foster an open-minded interpretation of participants’ experiences [84].

Along with this quantitative and thematic analysis, individual case reports were written for each participant, and all analysis is synthesized into the following Results section.

## Results

### Overview

**Figure 3.**
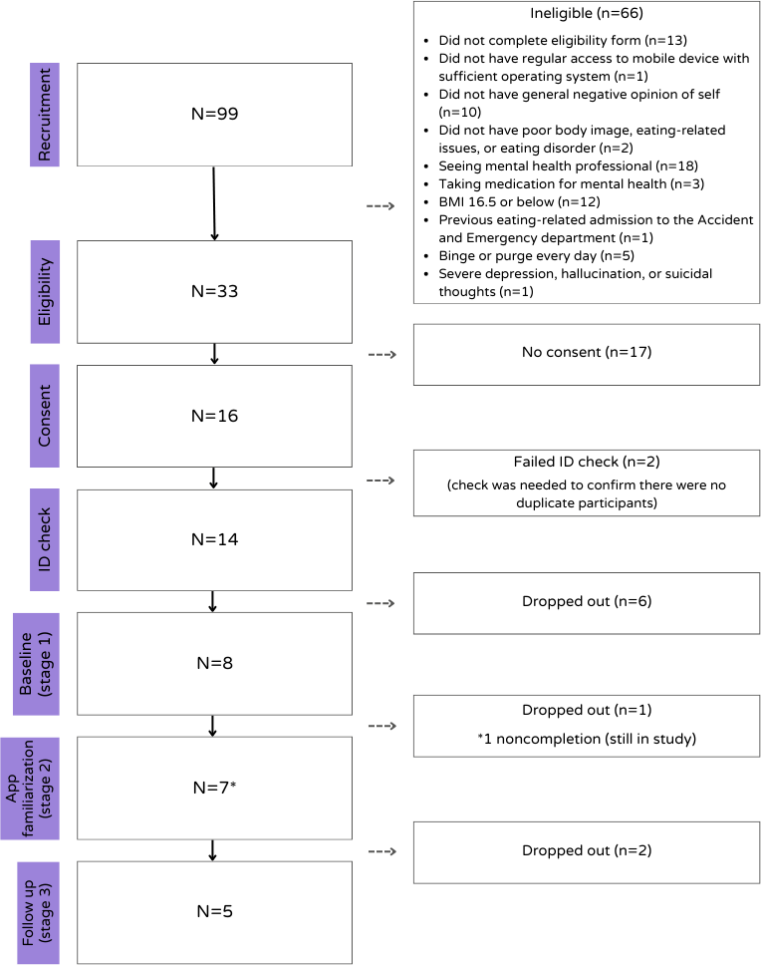
PRISMA participant flow diagram of the recruitment process for this preliminary case series pilot study of the Worth Warrior app for young people with low self-esteem and mild eating disorders. PRISMA: Preferred Reporting Items for Systematic Reviews and Meta-Analyses.

The recruitment for this study took place between September 2022 and July 2024. Due to the stringent eligibility criteria, 99 people registered their interest in taking part in this study, but 53 (54%) of these did not meet the eligibility criteria, and a further 13 people did not complete the eligibility form. Of the 33 who were eligible, 17 (52%) did not provide informed consent to take part, 2 (6%) did not pass the ID check (this was necessary to ensure all participants were unique), and a further 6 (18%) dropped out before commencing stage 1. Ultimately, 8 people entered this study; see Figure 3 for details of the recruitment flow.

### Participant Demographics

Of the 8 individuals who began the study, 5 (63%) completed stage 3 approximately 7 weeks later (a 38% attrition rate). One of these individuals did not complete the questionnaires at stage 2, but did complete stages 1 and 3, and so has been included in this preliminary evaluation.

These 5 participants (female=4, nonbinary=1) ranged in age from 19 to 25 (mean 22, SD 2.19) years. All 5 were White-British, with 3 in full-time employment, 1 in college, and 1 stay-at-home mother. See Table 1 for full details.

**Table 1.** Characteristics of the participants at baseline (stage 1) of this preliminary case series pilot study of the Worth Warrior app for young people with low self-esteem and mild eating disorders (N=5).

Characteristic	Values	
Gender, n (%)		
Women	4 (80)	
Nonbinary	1 (20)	
Country, n (%)		
England	5 (100)	
Ethnic background, n (%)		
White-British	5 (100)	
Employment or study status, n (%)		
Employed (full time)	3 (60)	
Student (full time or part time)	1 (20)	
Unemployed: stay-at-home mother	1 (20)	
Marital status, n (%)		
Single	2 (40)	
Married or cohabiting	2 (40)	
Other: engaged	1 (20)	
Age (years), n (%)		
19	1 (20)	
20	1 (20)	
23	2 (40)	
25	1 (20)	

### Overview

Table 2 details EDE-Q and Rosenberg Self-Esteem Scale scores, per study stage, for each participant; see Table 3 for cohort mean scores and SDs, per stage.

**Table 2.** Mean EDE-Q^a^, Rosenberg Self-Esteem Scale, and Worth Warrior app^b^ scores per participant, at each stage of this preliminary case series pilot study of the Worth Warrior app for young people with low self-esteem and mild eating disorders.

Scale and subscale		P9			P10			P12			P15			P17		
		Stage 1	Stage 2	Stage 3	Stage 1	Stage 2	Stage 3	Stage 1	Stage 2	Stage 3	Stage 1	Stage 2	Stage 3	Stage 1	Stage 2	Stage 3
EDE-Q, mean																
Global score		2.8	N/A^c^	0.5	4.3	0.5	0.8	4.0	4.0	2.0	5.1	4.7	5.3	4.8	4.8	5.2
Restraint		2.0	N/A	0.4	0.6	0.0	0.0	1.2	2.8	0.2	4.0	4.4	4.6	5.0	5.2	5.2
Eating concern		1.6	N/A	0.2	5.2	0.4	0.0	4.0	3.4	1.2	5.8	5.0	5.6	2.8	2.8	4.0
Shape concern		3.4	N/A	0.5	5.9	0.9	1.6	5.5	4.8	3.3	5.5	4.9	5.9	5.9	5.8	5.9
Weight concern		4.4	N/A	1.0	5.6	0.8	1.4	5.4	5.2	3.2	5.2	4.6	5.0	5.4	5.4	5.8
Behavior frequency		1.2	N/A	0.0	1.7	0.0	0.0	2.0	0.5	0.8	1.2	1.5	1.8	0.5	0.5	1.0
Rosenberg, mean (subscale N/A)		13	N/A	20	3	12	17	1	3	9	7	12	10	9	10	6
App (rating scales only)^b^, mean																
Usability		N/A	N/A	5.0	N/A	5.0	5.0	N/A	4.5	3.5	N/A	2.5	4.0	N/A	5.0	3.5
Acceptability		N/A	N/A	5.0	N/A	5.0	5.0	N/A	3.5	3.5	N/A	3.5	3.5	N/A	5.0	2.5
Safety		N/A	N/A	5.0	N/A	5.0	5.0	N/A	3.7	3.0	N/A	3.3	2.7	N/A	3.0	3.3

a EDE-Q: Eating Disorder Examination Questionnaire.

b This user questionnaire was developed for this study; each subscale is scored by averaging total ratings of the corresponding rating scales, resulting in a maximum score per subscale of 5. However, this does not take into account users’ qualitative responses, which are addressed in the main body.

c N/A: not applicable.

**Table 3.** Mean scores and SDs, per study stage, for the scales and subscales used in this preliminary case series pilot study of the Worth Warrior app for young people with low self-esteem and mild eating disorders.

Scale and subscale		Stage 1		Stage 2		Stage 3	
		Mean (SD)	n	Mean (SD)	n	Mean (SD)	n
EDE-Q^a^							
Restraint		2.56 (1.68)	5	3.10 (1.99)	4	2.08 (2.31)	5
Eating concern		3.88 (1.54)	5	2.90 (1.65)	4	2.20 (2.22)	5
Shape concern		5.23 (0.94)	5	4.06 (1.88)	4	3.43 (2.18)	5
Weight concern		5.20 (0.42)	5	4.00 (1.87)	4	3.28 (1.90)	5
Global score		4.22 (0.78)	5	3.52 (1.75)	4	2.75 (2.10)	5
Rosenberg	—^b^	6.60 (4.27)	5	9.25 (3.70)	4	12.40 (5.24)	5
App ratings							
Usability		—	—	4.25 (1.03)	4	4.20 (0.68)	5
Acceptability		—	—	4.25 (0.75)	4	3.90 (0.97)	5
Safety		—	—	3.75 (0.76)	4	3.80 (1.00)	5

a EDE-Q: Eating Disorder Examination Questionnaire.

b Not available.

### Baseline (Stage 1)

**Figure 4.**
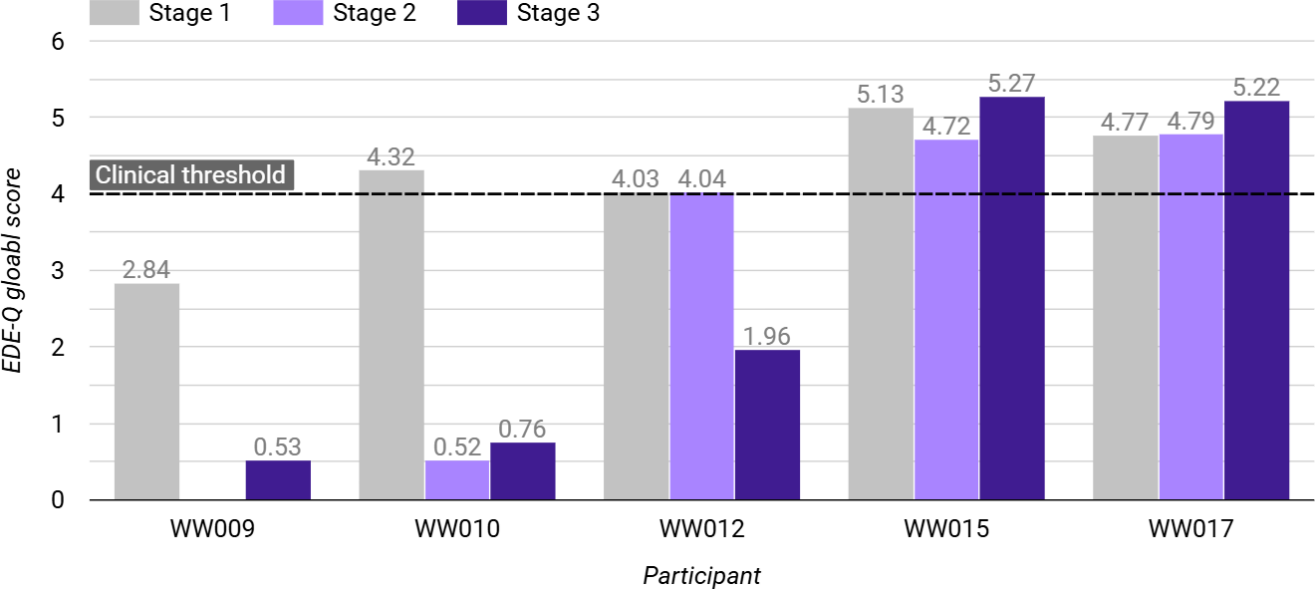
Participants’ global EDE-Q scores at each stage of this preliminary case series pilot study of the Worth Warrior app for young people with low self-esteem and mild eating disorders. The clinical threshold of EDE-Q scores ≥4 is indicated by the dashed line. EDE-Q: Eating Disorder Examination Questionnaire; WW: Worth Warrior.

At the point of commencing this study, all 5 participants were classed as either a “healthy” weight (n=3, 60%) or “overweight” according to their BMI, and all expressed current methods of coping with low self-worth that were either nonexistent or maladaptive (such as overreliance on family and friends or starving oneself in response to low self-worth). All indicated problematic attitudes and behaviors toward their eating, 4 of whom scored above the clinical threshold on the EDE-Q, with the remaining participant (P9) still scoring well above their (nonclinical) population mean (average EDE-Q global score=4.2, SD 0.8, range=2.8 to 5.1; clinical threshold≥4; [72]); see Figure 4.

All participants also indicated at least some elements of restraint, shape concern, eating concern, and weight concern, with 3/5 (60%) indicating their biggest problem as shape concern. Behaviorally, all participants reported instances of exercising compulsively within the past month, and all but one (P17) reported instances of feeling like they had lost control over their eating.

**Figure 5.**
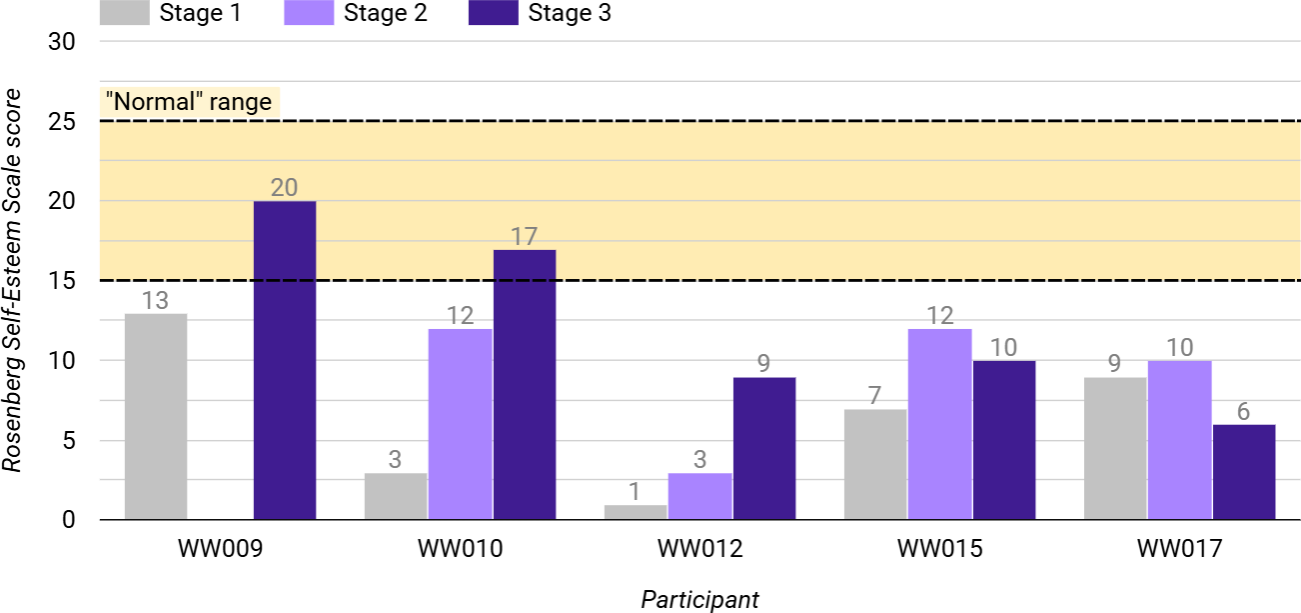
Participants’ Rosenberg Self-Esteem Scale scores at each stage of this preliminary case series pilot study of the Worth Warrior app for young people with low self-esteem and mild eating disorders. The range of scores considered to indicate “normal” levels of self-esteem is indicated between the dashed lines. WW: Worth Warrior.

Additionally, all indicated moderately low to extremely low levels of self-esteem (average Rosenberg Self-Esteem Scale score=6.6, SD 4.27, range=1 to 13; “low” self-esteem≥15); see Figure 5.

### Postfamiliarization Phase (Stage 2)

Having been introduced to the Worth Warrior app, participants spent an average of 12 (SD 7.66) days in the post–app familiarization phase (stage 2). Most (4/5, 80%) used it on an iPhone, with the remaining participant using an Android phone. Excluding P9, for whom there is no postfamiliarization data, during this phase, all participants reported using the Worth Warrior app fairly infrequently (either several times a week: 1/4, 25%; weekly: 2/4, 50%; or less than once a week: 1/4, 25%); and most (3/4, 75%) used it in the evenings or late at night, with the remaining participant using it at any time.

At the check-in at the end of this study stage, outcomes were mixed. A total of 2 participants had maintained their above–clinical threshold global EDE-Q scores from stage 1, while 2 had improved their attitudes and behaviors (ie, demonstrated decreased EDE-Q global scores, one of −0.4 points and the other of 3.8 points). One of these participants (P10) illustrated a change from above to below the clinical threshold.

Regarding EDE-Q subscales, all participants indicated at least a little improvement in their shape concern by this stage (average subscale decrease=1.6 points, SD 1.96, range of decrease=0.13 to 5.00), while restraint appeared to have slightly increased for 3/4 (75%) participants (average increase=0.73 points, SD 0.62, range=0.20 to 1.60). Eating concern and weight concern decreased for all but 1 participant (P17), for whom it stayed the same as at stage 1 (average decrease=1.97 points, SD 2.01, range=0.20 to 4.80).

Behaviorally, 2/4 (50%) participants indicated reductions in their behavior frequency (average decrease=1.6, SD 0.1, range=1.50 to 1.67), one remained the same (P17), and one showed a minor increase (+0.3; P15). Additionally, 2 participants reported no instances or only 1 instance of some of the eating disorder–related behaviors that they had engaged in regularly before starting this study. One participant (P10) also said that the app helped them to contact their mother about their difficulties with eating and body image.

In terms of self-esteem, all participants (4) also indicated increases in their self-esteem at this stage compared to the first (average increase in Rosenberg Self-Esteem Scale score=4.25 points, SD 3.1, range=1 to 9).

### Follow-Up (Stage 3)

Participants then spent an average of 40 (SD 1.6) days, or approximately 5 and a half weeks, using the Worth Warrior app in the intervention phase. During this time, the reported usage pattern was largely similar to that observed for stage 2, with no one using it every day and the majority (3/5, 60%) using it less than once per week, and most (4/5, 80%) using it either in the evening or late at night.

From stage 1 to 3 (baseline compared to postintervention), 3/5 (60%) participants indicated improvements in their eating disorder symptoms (average EDE-Q global score decrease=2.6 points, SD 0.65, range of decrease=2.06 to 3.56), with 2 moving from above to below the clinical threshold and 1 further reducing their subthreshold score from the beginning of this study; Figure 4. The formula by Jacobson and Truax [85], 1991, for the Reliable Change Index (RCI) was used to determine whether these improvements could be considered instances of reliable change, as opposed to expected fluctuations due to measurement unreliability. In all 3 cases, participants’ standardized RCI values for their EDE-Q global score reductions (range=−5.73 to −10.03) well exceeded the significance threshold of ±1.96, suggesting that these were statistically reliable changes at a 95% CI.

The remaining 2/5 (40%) participants showed very mild increases in their EDE-Q global scores (average increase=0.3, SD 0.2, range of increase=0.14 to 0.45), both of whom were already above threshold. Furthermore, neither of these EDE-Q increases was found to be reliable according to their standardized RCI values (range=0.57 to 1.15).

Additionally, 60% (3/5) of participants demonstrated reductions in all 5 of the EDE-Q subscales, with the largest reduction (−5.2 points) occurring for P10’s eating concern, which scored 0.0 by stage 3. Eating concern and weight concern reduced in all but 1 (P17) of the participants (4/5, 80%; average subscale reduction=2.5, SD 1.7, range of reductions=0.2 to 5.2), and restraint, shape concern, and behavior frequency reduced in 3/5 (60%; average subscale reduction=1.84 points, SD 1.1, range of reductions=0.6 to 4.25).

Behaviorally, 3/5 (60%) participants reported no instances of at least one of the eating disorder–related behaviors that they reported engaging in before starting this study, and 2 participants reported that the app helped them to contact someone for support, with one of these (P17) specifying contacting a mental health lead at their work.

Furthermore, 80% (4/5) of participants demonstrated increased levels of self-esteem from stage 1 to 3 (average increase=8 points, SD 3.94, range of increase=3 to 14), with 2 demonstrating a move from “low” self-esteem at baseline to the “normal” range by stage 3; Figure 5. The remaining participant (P17) illustrated a small decrease (−3 points) compared to their baseline.

### Usability, Acceptability, and Safety

#### Overview

The user questionnaire, delivered at the end of stages 2 and 3, facilitated insight into participants’ experiences with the Worth Warrior app over the total 7 weeks of this study, with a particular focus on perceived usability, acceptability, and safety.

#### User Ratings Following App Familiarization

Overall, the app was rated fairly favorably by participants at stage 2, receiving an average rating of 4.3/5 for both usability (SD 1.03, range=2.5 to 5.0) and acceptability (SD 0.75, range=3.5 to 5.0). In comparison, its safety was rated lower overall, at an average of 3.8/5 (SD 0.76, range=3.0 to 5.0). This predominantly came from 3/4 (75%) participants scoring 3/5 on some of the questions regarding whether the app helped reassure them when their self-worth was low or reduced negative self-evaluation.

Furthermore, the average usability scores varied the most at this stage, in which P15 rated a 3/5 for the app’s ease of use and 2/5 for how smoothly it ran (though reported no technical issues), while the remaining 3 participants rated usability at either 4.5 or 5 out of 5.

#### User Ratings Following the Intervention Phase

Following 6 weeks of use, the Worth Warrior app was again rated fairly favorably overall at stage 3, with the cohort rating its usability at an average of 4.2/5 (SD 0.68, range=3.5 to 5.0) and its acceptability at 3.9/5 (SD 0.97, range=2.5 to 5.00). Moreover, 2/5 (40%) participants rated both these constructs at 5/5, with 1 saying there was nothing that they did not like.

Two participants specified the “change” sections of the app as being the most useful for them, another 2 said the journal, and the remaining participant specified the psychoeducation information. This is reflected in participants’ qualitative answers regarding their preferences, in which 2 key subthemes were the active and interactive elements of the change activities and the facility for self-insight provided by the journal and self-monitoring section; this is discussed in further detail below. P12 reported finding the app a little harder to use than before, while P15 found it a little easier. Overall, however, participants’ usability ratings hovered at around 4/5, with relatively little variability.

Conversely, the average acceptability score had decreased slightly from 4.25 at stage 2 (−0.35/5). This came from a reduction in P17’s scores for this subscale, in which they rated lower satisfaction with the app and found it less useful than before. This aligns with this participant’s qualitative responses, in which they expressed dissatisfaction with some aspects of the app’s design (namely, requiring users to initiate their own use and activities), which is further discussed below. There was also greater variability in participants’ acceptability scores this time compared to the stage 2 check-in, ranging from 2.5/5 for P17 to 5.0 for P9 and P10.

In terms of safety, like at stage 2, the app was rated lower overall for its safety at stage 3 in comparison to the other constructs, at an average of 3.8/5 (SD 1.00, range=2.7 to 5.0). Additionally, as with usability scores, the cohort’s average rating for the app’s safety remained largely unchanged from stage 2. There was also a moderate amount of variability in participants’ scores for the app’s safety, ranging from 2.7/5 for P15 to 5/5 for P9 and P10.

P12 and P15 reported minor decreases in their average safety subscale scores compared to stage 2 (both −0.67/5). For P12, they were ambivalent about the app’s effectiveness in reassuring them when their self-esteem was low or in reducing negative self-evaluation. Both P15 and P17 were also ambivalent about the app helping to reassure them and were unconvinced (scoring 2/5) about it reducing negative self-evaluation.

See Multimedia Appendix 5 for a breakdown of responses to the safety subscale of the user questionnaire at stages 2 and 3.

Finally, at the end of this study, 2/5 (40%) participants gave the app the maximum score for all 3 categories (usability, acceptability, and safety).

#### Qualitative Findings

**Figure 6.**
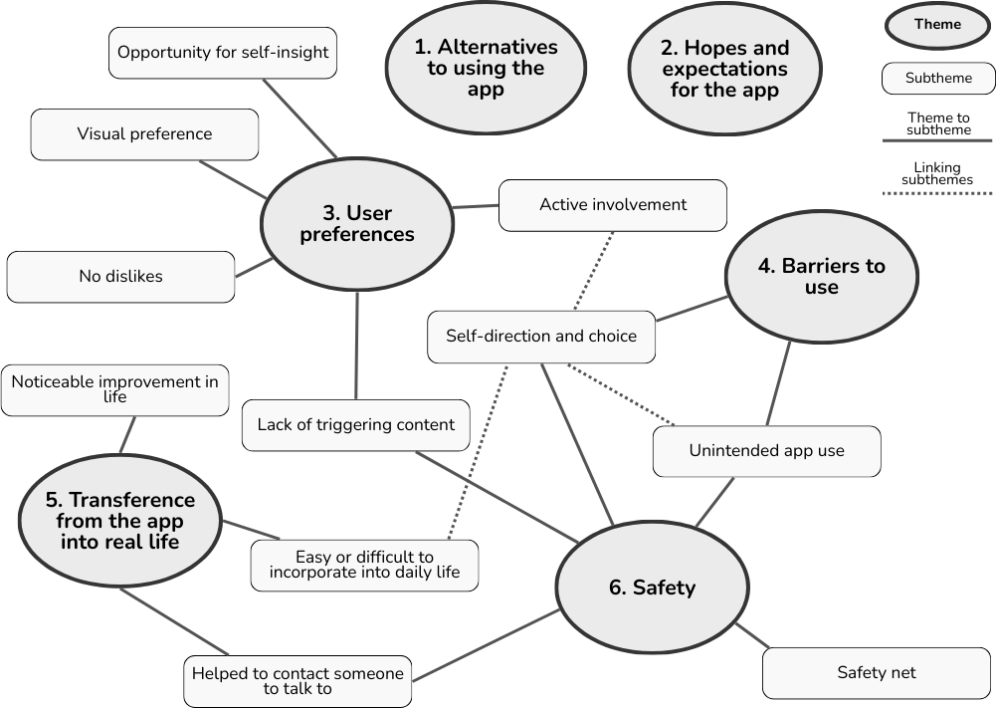
Thematic map of the themes and subthemes identified from qualitative responses collected in this preliminary case series pilot study of the Worth Warrior app for young people with low self-esteem and mild eating disorders.

Six key themes were identified from the qualitative data derived from responses to the user questionnaire, along with associated subthemes; Figure 6. These were (1) alternatives to using the app, (2) hopes and expectations for the app, (3) user preferences, (4) barriers to use, (5) transference from the app into real life, and (6) safety.

These are discussed below. The direct quotes from participants used throughout have been selected to provide a balance between clearly exemplifying the subthemes and themes and representing a balance of all participants.

#### Hopes and Expectations for the App

Before starting the study, participants’ expectations for the Worth Warrior app had been varied. A total of 2/5 (40%) participants had anticipated that the app would facilitate self-insight and reflection, while another 2 participants had expected a step further, thinking using it would help boost their confidence and reduce negative thinking.

*[I was expecting a] chance to consider my opinions around my self worth.*

[P17]

*To help me help myself deal with my feelings and thoughts, so hopefully I wouldn’t need to rely on other people as much.*

[P9]

Some (2/5, 40%) had comparatively low expectations (“not much, just a journal” [P15]).

Additionally, some participants (2/5, 40%) had also been somewhat unsure or unclear about what to expect, such as P9: “although I had expectations of what it could do for me, I didn’t have expectations about what the app would do to achieve it.” Interestingly, one of these participants (P12) stated that they did not know what they had expected of the app, though in later responses revealed a preconception that it would have involved inputting BMI or monitoring weight, which they were then glad to find it did not include as they would have found it triggering.

By the end of stage 3, most participants (3/5, 60%) stated that the app had met their expectations, with the remaining 2 participants saying it had either “helped in some ways” (although they had not expressed any specific expectations) or had “not massively” met their expectation of facilitating self-insight.

*[The app helped] more than I expected- with confidence, self respect and thought, changing perspective.*

[P10]

#### Alternatives to Using the App

A total of 3/5 (60%) participants mentioned having no other support available to them before entering this study. For example, P17 said that a *“*lack of available help from local GP [General Practitioner]*”* had led them to try using the Worth Warrior app, and P12 said that normally to manage low self-worth, they would just “deal with it on [their] own.” Additionally, 3 participants mentioned usually engaging in maladaptive behaviors, such as restriction and negative thinking (2/5, 40%) or a self-professed overreliance on other people (1/5, 20%).

*[Normally, to manage low self-worth I would engage in] bad habits, think about food and failure. Just overthinking and doing nothing.*

[P10]

Of the 2 participants who did not directly mention typically engaging in maladaptive behaviors to manage low self-worth, 1 participant said that they had “no other support in place” and expressed a desire for change (P17), and the other participant said they would talk with friends (P15).

*[I feel] that something has to change with my current eating situation.*

[P17]

#### User Preferences

##### Active Involvement

Most participants (4/5, 80%) mentioned that they valued the active involvement required in the app, in which activities are interactive and involve user input. This was especially so for the activities involving cognitive restructuring, and particularly the “change the way I feel about my body” activities, which were mentioned as favorite activities by 3/5 (60%) participants.

Additionally, P17, when asked about the features they liked using most, expressed liking the immediate support afforded by the interactive process of this section of the app:

*Changing the way I view my body gave me immediate help with strong self-perceptions.*

[P17]

Furthermore, 2/5 (40%) participants had said that they were hoping the app would provide an opportunity for self-reflection and to assess their own habitual thought processes, which may relate to this general preference of most participants for the cognitive restructuring activities.

*I liked the change your perspective part as it made you think in a more positive way instead of the first thought… thinking is it factual or a thought.*

[P10]

Another participant also said that they most liked the “change” features in general:

*I like [the Change features] because it’s more personalised and it helps me to keep that frame of mind outside the app.*

[P9]

Interestingly, however, some participants (2/5, 40%) expressed dislikes that relate to the level of active involvement asked of users, in contradiction to the above, instead desiring something more prescriptive. This is discussed later as its own subtheme within “barriers to use.”

##### Opportunity for Self-Insight

*[I wanted a] chance to consider my opinions around my self-worth.*

[P17]

As mentioned, 2/5 (40%) participants referenced a desire for facilitating self-reflection and insight with the app, which is also reflected in the fact that 2 other participants mentioned that they liked using either the journal or self-monitoring features.

*I liked monitoring myself through the journal.*

[P12]

##### Prompts

One participant (P17) mentioned liking the prompts in the app that help the user determine what they might want to say throughout the process of cognitive restructuring activities.

##### Lack of Triggering Content

One participant (P12), as mentioned, said that they were glad that the app did not include BMI or weight monitoring, as they had expected it might, as they would have found this triggering.

##### Visual Preferences

Lastly, P9 expressed that they did not like how the app was organized visually, in which each section is presented as a tab along the bottom of the screen. This was the only visual preference expressed by any of the participants.

Finally, 1/5 (20%) of participants said that there was nothing about the app that they did not like.

### Barriers to Use

#### Self-Direction and Choice

A concept that was continually identified in the data was the sense of a difficult and sometimes variable balance between user autonomy and guidance. As mentioned, several participants said that they valued the active involvement of app activities and personal configurability of the app, and P9 had also stated that they hoped the app would increase their own agency in managing their well-being: *“*[I was expecting the app to] help me help myself deal with my feelings and thoughts, so hopefully I wouldn’t need to rely on other people as much.*”*

However, 2/5 (40%) participants suggested that the app afforded perhaps too much independence and agency from them at times. In particular, P17 stated that they wished the app would send reminders to prompt use, and also to specify what to do in the app each day, rather than selecting it themselves.

*Generally I found it difficult to find the motivation to use the app without something specific to complete every day. … [I didn’t like] having to prompt myself to open it- reminders would be good.*

[P17]

They also said that the abundance of choice for what to work on in the app was “overwhelming” and “not what I want when I’m looking for help.”

For P12, they stated that they found difficulty with progressing to a more positive or neutral thought about themselves when the app prompted this: *“*I struggled with the complimenting myself after saying what’s wrong. I’m not in the head space to say what I like about myself when I’ve just said I dont.*”* Relatedly, this participant also said that they had set themselves maladaptive goals in the free-text fields in the app when they were prompted to set a positive goal, demonstrating a potential issue in giving users the freedom to specify their own targets.

#### Unintended App Use

This participant (P12), therefore, exemplifies another barrier, which was the unintended use of the app. Using the free-text fields in app features to enter unhelpful comments was, of course, unintended, and this participant also appears to have misunderstood the process in the cognitive restructuring activities in which the user is guided to replace a negative, automatic thought about themselves with one that is more neutral or objective. The prompts in the app state, “Can you think of a more accurate and factual thought?” along with examples; however, P12 appears to have misinterpreted this as a direction to input positive or complimentary thoughts about themselves, which they have then found difficult to do, and potentially distressing.

### Transference From the App Into Real Life

A total of 3/5 (60%) participants mentioned that the app had helped to produce noticeable improvement in their lives, such as helping them to contact someone to talk to about their mental health where needed (2/5, 40%), increasing their confidence or reducing negative thinking (3/5, 60%), or achieving their hope of relying on others less (1/5, 20%).

*I like [the Change features] because it’s more personalized and it helps me to keep that frame of mind outside the app. … With the app there I could casually use it without needing much thought.*

[P9]

While P9 mentioned that the app facilitated use in their daily life without much effort, the opposite was found for P17. As mentioned, this participant found that they struggled to either remember to or find motivation to use the app and wanted reminders and prescribed tasks to help with this, suggesting that they struggled to integrate the app into their daily life.

### Safety

Additionally, most participants (4/5, 80%) mentioned elements relating to app safety in their qualitative responses to the user questionnaire. In terms of positives, 1 participant mentioned (as referenced earlier) that they were glad that the app did not include content they would find triggering, and (as also referenced earlier) 2/5 (40%) participants said the app helped them to contact someone, such as their mental health lead at work (P17).

For negatives relating to app safety, some have also already been covered elsewhere: P17 said that the abundance of choice in the app was *“*overwhelming*”* for them and *“*not what I want when I’m looking for help*”* and P12 said that they were not prevented from writing maladaptive goals or content in their journal, given the free-text fields provided there. In this sense, the earlier subtheme of unintended app use can also apply here.

Finally, 1 participant (P15) said that they did not like the safety net in the app, which is an area where the user inputs things to do to help themselves and people to contact when they need extra support. Interestingly, this was the only time the safety net was mentioned by participants, and unfortunately, this participant did not expand on why they did not like it.

## Discussion

### Principal Findings

This case series is the first to offer insight into 5 young people’s experiences of using the Worth Warrior app to manage low self-esteem and mild eating difficulties. After 7 weeks of app use, outcome measures at follow-up showed reliable improvements in eating disorder symptomatology in 3/5 cases, and increases in self-esteem in 4/5 cases. Participants gave detailed feedback suggesting the app was acceptable, usable, and safe for some, though less so for others, and possible reasons for this, along with the outcome measures, are discussed below. Overall, the app shows considerable promise in providing an accessible standalone tool for managing mild eating disorder symptoms and low self-esteem in young people; however, this may be dependent upon particular factors such as symptom severity, and study limitations should be noted when interpreting these findings.

While this preliminary case series of a small sample means that no causal conclusions or attempts to generalize findings can be made, it provides a valuable first step in the form of in-depth insight into the app’s potential to be an effective and acceptable early intervention tool for young people, how it may most successfully be used, and cases in which it may not be best placed. It also identifies ample areas for further research.

Furthermore, case series offer the advantage of greater external validity than, say, randomized controlled trials [86], potentially therefore offering valuable insight for use in real-life settings. However, future research with larger and more diverse samples would be necessary to help determine generalizability.

### Potential Effectiveness

A total of 3 participants showed statistically reliable improvements in eating disorder symptomatology at follow-up, in which 2 participants had moved from above to below the clinical threshold of the EDE-Q, and 1 participant further reduced their subthreshold baseline score. Pre- and postintervention data for these 3 participants suggest that the app has potential to be effective across all 4 subscales of the EDE-Q (eating concern, weight concern, shape concern, and restraint) as well as behaviorally, and aligns with existing literature indicating that traditional eating disorder treatments, such as CBT-E, can be effectively delivered digitally [66]. It is also promising to note that, at follow-up, these 3 participants reported no instances of at least one of the eating disorder–related behaviors that they reported engaging in before starting the current study. Research with a larger sample and control group would be an important next step to clarify whether these observed changes are generalizable and can be confidently attributed to the use of the Worth Warrior app.

Conversely, the remaining 2 participants exhibited comparatively mild increases in EDE-Q global scores from stages 1 to 3, which, owing to the corresponding RCI values not exceeding the significance threshold, is likely from the scale’s inherent variability rather than any meaningful change in symptomatology. It may therefore be more appropriate to say that these remaining 2 participants did not exhibit any meaningful change in their pre- vs postintervention eating disorder symptomatology. Additionally, one of these participants also did not show improvements in self-esteem.

Further research would therefore also be beneficial to clarify reasons for these mixed results, as well as whether any potential increase in symptomatology is a result of app use rather than measurement variability, and who would be most likely to benefit from the app. For example, a longer period of use may be necessary for individuals with greater eating disorder symptomatology before positive change can be observed; longitudinal research would therefore be helpful to ascertain whether individuals with higher EDE-Q starting scores can see further benefit from the app over a longer time frame than that used in this study, and would also provide insight into whether any improvements can be maintained over time.

An alternative explanation is that the app may not be suitable or as effective as a standalone intervention for individuals with moderate to severe eating disorder symptomatology, as the 2 participants who did not show improvements at follow-up were those with the highest baseline scores (global scores of 4.8 and 5.1), and similarly the 2 participants who showed the greatest benefit were among those with the lowest starting symptomatology (global scores of 2.8 and 4.3). This study did not clarify whether participants had early-stage or more established disorders, as it did not obtain information regarding participants’ illness duration or time of onset, or whether they had previously been treated for an eating disorder. This information could have been helpful to provide insight into whether the app was more suited for individuals with early-stage, mild disorders vs those that are more established. The Worth Warrior app does indeed position itself as a tool for subclinical or early-stage disorders, and it may be argued that the present results therefore have limited applicability to populations classified as “early stage.” This identifies another avenue for further study, which would be useful to clarify this point.

Additionally, 4/5 participants showed increased self-esteem by stage 3, with 2 participants moving from “low” to “normal” levels. Interestingly, this includes those with the lowest baseline self-esteem levels of the cohort, suggesting that a user’s level of self-esteem may not impact the effectiveness of the app in the same way that their severity of eating disorder symptomatology may do, as proposed above.

### Usability, Acceptability, and Safety

The Worth Warrior app was well received by the cohort in general (though more so for its usability and acceptability than safety, as further discussed below), and the interactive features offering immediate support, cognitive restructuring exercises, and journal facilities were particularly favored. Additionally, 3/5 participants also said that it met or exceeded their expectations.

That said, some participants did express dislikes, and, as mentioned, participants had more mixed experiences regarding the app’s safety than for its usability or acceptability. These all align with the variations in outcome measures at follow-up as discussed earlier, such that those with the highest starting eating disorder symptoms were those who expressed the greatest barriers to use or dislikes with the app, and were also those who gave the lower ratings for the app’s safety and reported the least (or no) improvement in outcome measures. This does make theoretical sense; people with eating disorders are a group often known to resist change or treatment and display low motivation to engage in interventions [60,87]. While an app intervention may be desirable in this respect for some, allowing the person to feel in control of their engagement and treatment, for others—and likely those with greater symptomatology [87]—the burden to initiate one’s own engagement with the app may prove to be a barrier, and this has been particularly exemplified in P17’s reported experience. Consequently, it is not surprising that any difficulties in engaging, and engaging safely, with the app could transfer into its effectiveness as well as negatively impact the experiences of these users.

Similarly, eating disorder symptomatology can be associated with persistent negative self-talk or emotional dysregulation, which, such as in the case of P12, may be a reason why some users could struggle to engage with the directions of the app to input neutral self-statements or productive journal entries, in contrast to the directions of the app, thus compromising the app’s safety [88,89]. This is a further reason why the Worth Warrior app may therefore be best suited as a standalone tool only for mild presentations and should be used with clinician guidance and supervision in moderate to severe cases.

Additionally, these findings highlight areas for improvement in the app, such as greater guidance where necessary to ensure appropriate activity use (perhaps clearer prompts and instructions to input only neutral or positive statements), or even limitations on user-inputted text. Offering a more prescriptive approach to the activities, instead of requiring the user themselves to decide, could also be helpful for those individuals who can find the abundance of choice overwhelming, as in P17’s case, or incorporating regular reminders to use the app via device notifications—something that may also alleviate some of the dropout issues often found in digitally delivered eating disorder interventions [66].

It is promising to note that using the app encouraged 2 of the participants to contact people in their lives for additional support. This includes P17, who otherwise indicated no benefit in their eating disorder symptomatology or self-esteem, though they did express that “‘Change the way I view my body’ gave me immediate help with strong self-perceptions.” This is a promising finding regarding app safety, particularly for those with higher starting symptoms.

It is also interesting to note that 3/4 of applicable participants’ restraint, as measured by the EDE-Q, increased following app familiarization. It may be the case that, at first, engaging with the app can produce a greater awareness of one’s restraint behaviors and preoccupation with these, which further app use may then help to alleviate. This is a further reason why the app should be used with clinician guidance for stronger presentations to monitor this potential adjustment phase.

The findings, therefore, indicate that the app does show potential, particularly for mild presentations, though further clarification of appropriate target populations and expected timeframes for seeing meaningful change would be beneficial, and care should be taken to ensure the app is used appropriately and safely, particularly for individuals with greater symptom severity. This may require clinical supervision, particularly for anything greater than mild presentations, and/or clearer guidance in problem areas of the app to ensure users are suitably understanding what is being asked of them, and that they are engaging appropriately and safely. The app may therefore be best placed as an early motivation-enhancing tool when self-guided, with possible potential for early intervention alongside guided support.

### Study Limitations and Areas for Further Research

The limited diversity of the present sample should, however, be noted, as should the possibility of recruitment bias in which opportunity sampling, self-selection, and attrition may have resulted in a biased sample. This study sought to ensure ethnic diversity by recruiting participants from Birmingham (United Kingdom) and surrounding areas known for their high ethnic mix, as well as both the user and research advisory groups, including individuals from a range of cultural backgrounds, who contributed to shaping this study’s design, protocol, and questionnaires to ensure cultural sensitivity and relevance. Despite this, relatively few candidates from minority backgrounds commenced this study (n=2, one of Black-African and one of Asian/Asian-British ethnicity), and none completed all stages (additionally, the ethnicity of ineligible study applicants is unknown). It may be the case, therefore, that few candidates from minority backgrounds applied, or that many of those who did did not meet inclusion criteria, such as those who were already engaged with services or presented with significant symptoms beyond the study’s remit; however, this is purely speculative.

Accordingly, future research should further explore barriers to participation among ethnically diverse communities. Potential strategies include (1) collaborating closely with community and faith leaders to identify and address trust or stigma-related barriers; (2) enhancing transparency about research aims and participant safeguards to build confidence in the process; and (3) using purposive sampling, directly engaging with underrepresented groups, and inviting them to participate.

These steps would help ensure future studies are more inclusive and representative of the population affected by eating disorders.

For example, with all participants being White-British, and all but 1 participant identifying as female, it is as yet unclear whether experiences of using the app and its effectiveness may differ depending on factors such as ethnicity, gender, location, or age. This is particularly so considering how eating disorders can vary in prevalence, symptomatology, treatment seeking, and treatment response, as well as in biological and psychosocial factors and vulnerabilities, across factors including gender, age, sexual orientation, culture, and race [90,91]. Indeed, the one nonbinary participant in this study did indicate lower app effectiveness than some other participants, suggesting that there may be differences in the app’s effectiveness depending on demographic factors; however, this would require future research to determine. This also includes whether results can generalize to younger and older age groups than those involved in this study, as well as whether the type of eating disorder or the presence of any comorbidities makes any difference in the intervention’s effectiveness.

Furthermore, the recruitment difficulties and high attrition rate of this study may also suggest that the app had low appeal or acceptability to a lot of the target population and may have resulted in a biased final sample consisting of individuals who already found the app particularly acceptable. Including reminders to use the app, as suggested by P17, may also help to reduce user dropout in this way. It is also possible that the materials used to recruit participants may have introduced bias; for example, the background image used in the recruitment flyer most prominently displays White individuals, which, given the all-White resulting sample of the study, may possibly have discouraged non-White individuals from applying.

Finally, follow-up research over a longer period of time would also be valuable to explore whether any effects of using the Worth Warrior app can be sustained over time and/or continue to improve with prolonged use. This is especially as there was variability in the times at which participants indicated change, such as P10, who was the only participant to show their greatest change at stage 2 rather than 3, or P15, who showed a dip in EDE-Q scores at stage 2 but a reversal of this by stage 3. Comparisons with alternative treatments (such as other digital interventions or face-to-face treatments) would also be valuable to more clearly ascertain the app’s value in relation to alternative treatment options. Lastly, research examining the app’s cost-effectiveness in both the treatment and prevention of eating disorders in young people would also be a valuable addition.

### Conclusion

#### Summary of Findings

This preliminary investigation provides early support for the feasibility and acceptability of delivering cognitive behavioral therapy for eating disorders (CBT-E) via a mobile app, Worth Warrior, to individuals presenting with mild eating disorder symptoms. Although the sample size was limited, findings indicate that multiple participants experienced improvements in self-esteem and eating disorder symptomatology and reported positive engagement with the intervention, and the app was largely well-received, particularly regarding its usability and acceptability. Notably, the “change” modules in the app, targeting the identification and modification of negative cognitions related to body image, were perceived as particularly beneficial in fostering cognitive and emotional shifts.

However, some participants found difficulty with the level of self-direction and independence required of using the app as a standalone tool and did not see improvement in outcomes at follow-up. Relatedly, user safety would likely be maximized by ensuring that individuals with more severe eating disorder presentations use it alongside clinician guidance, and it is recommended that the app be used as a standalone intervention tool only for those with mild symptomatology. Several areas of improvement were also identified for the app to encourage appropriate use and promote user safety and engagement.

As this was an exploratory pilot study, these findings should be interpreted as indicative rather than conclusive.

#### Limitations and Future Research

Given that only 5 participants completed this study, these findings should be interpreted with caution and regarded as exploratory, as the small sample size restricts both the generalizability and statistical power of the results. Additionally, the absence of a control group prevents conclusions regarding causality or comparison with standard CBT-E delivery methods. Future research should therefore aim to replicate and extend these results using larger, more diverse samples and rigorous study designs, including randomized controlled trials and longitudinal follow-up. Such investigations would help to clarify the efficacy, mechanisms of change, and sustainability of outcomes associated with app-based CBT-E interventions.

Nonetheless, the current findings underscore the potential of digital CBT-E tools such as the Worth Warrior app to enhance self-esteem and address core cognitive features of eating disorders at a mild stage, thereby contributing to accessible and preventative approaches within the continuum of eating disorder care.

## Supplementary material

10.2196/79770 Multimedia Appendix 1 Example advertisement used for recruitment in this study.

10.2196/79770 Multimedia Appendix 2 The full screening tool used in this study.

10.2196/79770 Multimedia Appendix 3 Screening questions and inclusion criteria for this preliminary case series pilot study of the Worth Warrior app for young people with low self-esteem and mild eating disorders.

10.2196/79770 Multimedia Appendix 4 Worth Warrior app user questionnaire.

10.2196/79770 Multimedia Appendix 5 A breakdown of responses to the safety subscale of the Worth Warrior app user questionnaire at stages 2 and 3 of this preliminary case series pilot study of the Worth Warrior app for young people with low self-esteem and mild eating disorders.

## Abbreviations

CBT-E: enhanced cognitive behavioral therapy
EDE-Q: Eating Disorder Examination Questionnaire
NHS: National Health Service
RCI: Reliable Change Index
