# Supplementary figures and images for: Preliminary Case Series of the Worth Warrior Mobile App for Young People With Low Self-Esteem and Mild Eating Disorders: Pre– and Post–Follow-Up Study
Source: JMIR Form Res. 2026 Jan 20;10:e79770. doi: 10.2196/79770 (PMC12818502; doi:10.2196/79770)

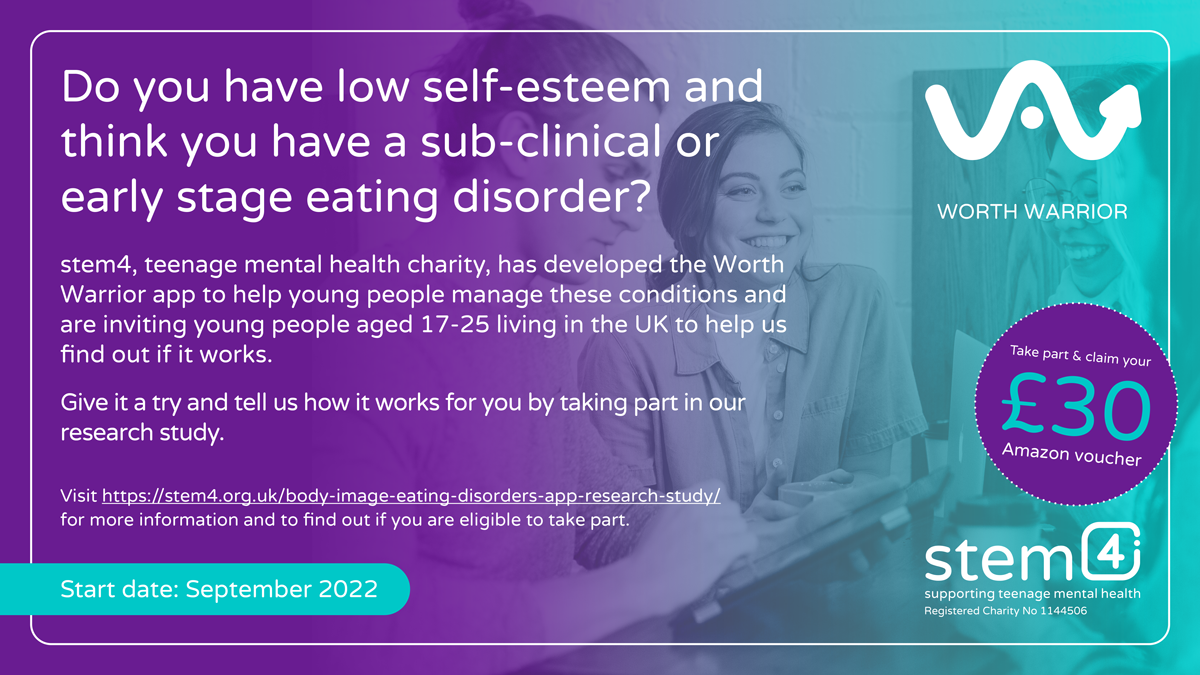

Supplement: Multimedia Appendix 1 — Example advertisement used for recruitment in this study. [file formative-v10-e79770-s001.png]
