# Supplementary material for: Preliminary Case Series of the Worth Warrior Mobile App for Young People With Low Self-Esteem and Mild Eating Disorders: Pre– and Post–Follow-Up Study
Source: JMIR Form Res. 2026 Jan 20;10:e79770. doi: 10.2196/79770 (PMC12818502; doi:10.2196/79770)
Supplement: Multimedia Appendix 2 — The full screening tool used in this study. [file formative-v10-e79770-s002.docx]

**Online Eligibility Screening Questionnaire**

(To be completed once online to determine eligibility status)

1. What country do you live in?

England

Northern Ireland

Wales

Scotland

Other (please specify)

1. What age are you in years?

17

18

19

20

21

22

23

24

25

Other (please specify)

1. Do you have regular access to an iOS device (e.g iPhone or iPad) running iOS v14 or higher or an Android device running Android v10 or higher?

Yes No

1. Do you have a generally negative opinion of yourself, are self-critical or place a negative value on yourself as a person?

Yes No

1. Do you have poor body image, eating-related issues, or an eating disorder?

Yes No

1. Are you currently seeing a mental health professional (not a GP, but a psychologist, psychiatrist or psychiatric nurse for example) for a mental health problem?

Yes No

1. Do you take any medication for a mental health problem?

Yes No

1. Do you have a body mass index (BMI*) of 16.5 or under? (*BMI is weight in Kg divided by height in meters squared or you can use this BMI calculator https://www.nhs.uk/live-well/healthy-weight/bmi-calculator/?

Yes No

1. Have you ever been admitted to Accident and Emergency for eating related problems?

Yes No

1. Do you binge or purge every day?

Yes No

1. Do you have any medical problems such as diabetes or a heart condition that needs to be monitored?

Yes No

1. Do you have severe depression, hallucinations or suicidal thoughts?

Yes No

1. Do you drink alcohol excessively or take any Class A drugs (for example, cocaine, speed, spice) on a weekly basis?

Yes No

What email address can we use to contact you?

………………………………………………………….

**Emergency numbers, information and support**

**In an emergency**

- If you are experiencing a mental health crisis visit Accident and Emergency (A&E) or call 999.

**Information, guidance and support**

- If you would like information or guidance for any mental health issues please follow this link: https://stem4.org.uk/wp-content/uploads/2020/06/Tesco-Booklet-Web-Version-2.pdf
- For advice and information and who to contact concerning eating disorders please follow this link: https://stem4.org.uk/wp-content/uploads/2019/09/Eating-Disorders-leaflet.pdf
- Or visit Beat (a national eating disorders charity) for help, advice and support: <https://www.beateatingdisorders.org.uk/get-information-and-support/>
- To talk to a counsellor for free you can call Childline: 0800 1111; or contact The Mix by phone:08088084994, or via this link: <https://www.themix.org.uk/> (which includes a free chat line)
- For practical advice on suicide prevention you can contact HopeLine UK on 0800 068 4141 (www.papyrus-uk.org)

**Thank you for completing this form.**
