# Supplementary material for: Preliminary Case Series of the Worth Warrior Mobile App for Young People With Low Self-Esteem and Mild Eating Disorders: Pre– and Post–Follow-Up Study
Source: JMIR Form Res. 2026 Jan 20;10:e79770. doi: 10.2196/79770 (PMC12818502; doi:10.2196/79770)
Supplement: Multimedia Appendix 3 — Screening questions and inclusion criteria for this preliminary case series pilot study of the Worth Warrior app for young people with low self-esteem and mild eating disorders. [file formative-v10-e79770-s003.docx]

| **Screening question** | **Inclusion criteria** |
| --- | --- |
| What country do you live in? | Live in the UK |
| What age are you in years? | Be age 17-25 |
| Do you have regular access to an iOS device (e.g iPhone or iPad) running iOS v14 or higher or an Android device running Android v10 or higher? | Have access to a device suitable for running the app sufficiently and for regular access to be able to use the app as and when needed |
| Do you have a generally negative opinion of yourself, are self-critical or place a negative value on yourself as a person? | Have low self-worth |
| Do you have poor body image, eating-related issues, or an eating disorder? | Have poor body image, eating-related issues, or a mild eating disorder |
| Are you currently seeing a mental health professional (not a GP, but a psychologist, psychiatrist or psychiatric nurse for example) for a mental health problem? | Not seeing a mental health professional for a mental health problem, to control for extraneous variables that may affect the dependent variables and to exclude individuals with likely more moderate to severe presentations. |
| Do you take any medication for a mental health problem? | Not taking any medication for a mental health problem, to control for extraneous variables that may affect the dependent variables |
| Do you have a body mass index (BMI*) of 16.5 or under? (*BMI is weight in Kg divided by height in meters squared or you can use this BMI calculator https://www.nhs.uk/live-well/healthy-weight/bmi-calculator/? | Not have a Body Mass Index (BMI) of 16.5 or under, as this was considered too severe of a case for independent use of the Worth Warrior app |
| Have you ever been admitted to Accident and Emergency for eating related problems? | Not been admitted to Accident and Emergency for eating-related problems, as this was considered to indicate too severe of a case for independent use of the Worth Warrior app and was also to exclude individuals with likely more moderate to severe presentations. |
| Do you binge or purge every day? | Not binge or purge every day, because these difficulties were considered too severe for independent use of the Worth Warrior app |
| Do you have any medical problems such as diabetes or a heart condition that needs to be monitored? | Not have any medical problems such as diabetes or a heart condition that needs to be monitored, as this was considered too severe of a case for independent use of the Worth Warrior app |
| Do you have severe depression, hallucinations or suicidal thoughts? | Not have severe depression, hallucinations or suicidal thoughts, as this was considered too severe of a case for independent use of the Worth Warrior app |
| Do you drink alcohol excessively or take any Class A drugs (for example, cocaine, speed, spice) on a weekly basis? | Not drink alcohol excessively or take any Class A drugs (for example, cocaine, speed, spice) on a weekly basis, to control for extraneous variables that may affect the dependent variables |
