# Supplementary material for: Preliminary Case Series of the Worth Warrior Mobile App for Young People With Low Self-Esteem and Mild Eating Disorders: Pre– and Post–Follow-Up Study
Source: JMIR Form Res. 2026 Jan 20;10:e79770. doi: 10.2196/79770 (PMC12818502; doi:10.2196/79770)
Supplement: Multimedia Appendix 4 — Worth Warrior app user questionnaire. [file formative-v10-e79770-s004.docx]

***Usability***

1. **What platform did you use for the WW app? (Please tick one)**

Android (smartphone)

IPhone

Other (please specify)

1. **How smoothly did the WW app run? (Please circle one)**

Very smoothly 5 4 3 2 1 Not smoothly at all

1. **How easy was the app to use?**

Very easy 5 4 3 2 1 Not easy at all

1. **Where there any technical issues when using the WW app?**

Yes

No (please describe)

1. **How often did you use the WW app? (Please tick one)**

More than once a day

Every day

Several times a week

Weekly

Less than once a week

1. **What time of day did you use the WW app? (Please tick all that apply)**

Morning

Afternoon

Evening

Late at night

Anytime

1. **When was the last time you used the app?**

Date:

***Acceptability***

1. **Which parts of the WW app did you use? (Please tick all that apply)**

- Change your story?
- Change the action?
- Too many behaviours?
- Restricted/reduced behaviours?
- Change the emotion
- Change your perception

**8a. Please rate how satisfied you were with the WW app:**

Very satisfied 5 4 3 2 1 Not satisfied at all

1. **Which of parts of the App did you find the most useful? (Please tick all that apply)**

- The change sections
- Safety net
- Social media and me
- Immediate help
- Information
- Self-monitoring
- Self-worth tracker
- My journal
- Activity tracker
- My goals

**9a. Please rate overall how useful you found the WW app to be for you:**

Very useful 5 4 3 2 1 Not useful at all

1. **What features did you like using most and why?**
2. **What features did you not like and why?**
3. **What were the circumstances that lead you to use the WW app?**
4. **What would you normally have done for managing low self-worth instead of using the WW app?**
5. **What were you expecting from the WW app?**
6. **How closely did the WW app meet those expectations?**
7. **Was there anything you expected from the WW app that it didn’t achieve?**
8. **Did you unload the WW app? (If so, please explain why)**

***Safety***

1. **Did the WW app help reassure you when your self-worth was low?**

Yes, a lot 5 4 3 2 1 No, not at all

1. **Did the WW app reduce negative self-evaluation?**

Yes, a lot 5 4 3 2 1 No, not at all

1. **Did the WW app increase negative self-evaluation when you used it?**

No, not at all 5 4 3 2 1 Yes, a lot

1. **Did the WW app help you to contact someone to speak to about your feelings of low self-worth and the impact this may have on your eating behaviours?**

Yes (Who did you contact – a friend, relative, health professional?)

No

**THANK YOU FOR COMPLETING THIS QUESTIONNAIRE**
