# Supplementary material for: Preliminary Case Series of the Worth Warrior Mobile App for Young People With Low Self-Esteem and Mild Eating Disorders: Pre– and Post–Follow-Up Study
Source: JMIR Form Res. 2026 Jan 20;10:e79770. doi: 10.2196/79770 (PMC12818502; doi:10.2196/79770)
Supplement: Multimedia Appendix 5 — A breakdown of responses to the safety subscale of the Worth Warrior app user questionnaire at stages 2 and 3 of this preliminary case series pilot study of the Worth Warrior app for young people with low self-esteem and mild eating disorders. [file formative-v10-e79770-s005.docx]

| Questions and responses | *Stage 2*  (N=4) | | *Stage 3*  (N=5) | |
| --- | --- | --- | --- | --- |
|  | *N* | *%* | *N* | *%* |
| **Did the Worth Warrior app help reassure you when your self-worth was low?**  1 (No, not at all)  2  3  4  5 (Yes, a lot) | 0  0  2  1  1 | 0  0  50  25  25 | 0  0  3  0  2 | 0  0  60  0  40 |
| **Did the WW app reduce negative self-evaluation?**  1 (No, not at all)  2  3  4  5 (Yes, a lot) | 0  0  2  1  1 | 0  0  50  25  25 | 0  2  1  0  2 | 0  40  20  0  40 |
| **Did the WW app increase negative self-evaluation when you used it? (reverse coded)**  1 (Yes, a lot)  2  3  4  5 (Not at all) | 0  0  2  1  1 | 0  0  50  25  25 | 0  0  2  0  3 | 0  0  40  0  60 |
| **Did the WW app help you to contact someone to speak to about your feelings of low self-worth and the impact this may have on your eating behaviours?**  Yes  No | 1  3 | 25  75 | 2  3 | 40  60 |
